# Discrimination of grade 2 and 3 cervical intraepithelial neoplasia by means of analysis of water soluble proteins recovered from cervical biopsies

**DOI:** 10.1186/1477-5956-9-36

**Published:** 2011-06-28

**Authors:** Kai-Erik Uleberg, Ane Cecilie Munk, Cato Brede, Einar Gudlaugsson, Bianca van Diermen, Ivar Skaland, Anais Malpica, Emiel AM Janssen, Anne Hjelle, Jan PA Baak

**Affiliations:** 1 Pathology Department, Stavanger University Hospital, Armauer Hansen Road 20, Stavanger, Norway; 2 Gynaecology Department, Stavanger University Hospital, Armauer Hansen Road 20, Stavanger, Norway; 3 The Gade Institute, University of Bergen, Bergen, Norway; 4 International Research Institute of Stavanger (IRIS), Stavanger, Norway; 5 Departments of Pathology and Gynecologic Oncology, The University of Texas M. D. Anderson Cancer Center, Houston, TX; 6 Department of Medical Biochemistry, Stavanger University Hospital, Armauer Hansen Road 20, Stavanger, Norway

**Keywords:** cervical intraepithelial neoplasia, CIN, proteomics, LTQ-Orbitrap, mass spectrometry

## Abstract

**Background:**

Cervical intraepithelial neoplasia (CIN) grades 2 and 3 are usually grouped and treated in the same way as "high grade", in spite of their different risk to cancer progression and spontaneous regression rates. CIN2-3 is usually diagnosed in formaldehyde-fixed paraffin embedded (FFPE) punch biopsies. This procedure virtually eliminates the availability of water-soluble proteins which could have diagnostic and prognostic value.

**Aim:**

To investigate whether a water-soluble protein-saving biopsy processing method followed by a proteomic analysis of supernatant samples using LC-MS/MS (LTQ Orbitrap) can be used to distinguish between CIN2 and CIN3.

**Methods:**

Fresh cervical punch biopsies from 20 women were incubated in RPMI1640 medium for 24 hours at 4°C for protein extraction and subsequently subjected to standard FFPE processing. P16 and Ki67-supported histologic consensus review CIN grade (CIN2, n = 10, CIN3, n = 10) was assessed by independent gynecological pathologists. The biopsy supernatants were depleted of 7 high abundance proteins prior to uni-dimensional LC-MS/MS analysis for protein identifications.

**Results:**

The age of the patients ranged from 25-40 years (median 29.7), and mean protein concentration was 0.81 mg/ml (range 0.55 - 1.14). After application of multistep identification criteria, 114 proteins were identified, including proteins like vimentin, actin, transthyretin, apolipoprotein A-1, Heat Shock protein beta 1, vitamin D binding protein and different cytokeratins. The identified proteins are annotated to metabolic processes (36%), signal transduction (27%), cell cycle processes (15%) and trafficking/transport (9%). Using binary logistic regression, Cytokeratin 2 was found to have the strongest independent discriminatory power resulting in 90% overall correct classification.

**Conclusions:**

114 proteins were identified in supernatants from fresh cervical biopsies and many differed between CIN2 and 3. Cytokeratin 2 is the strongest discriminator with 90% overall correct classifications.

## Background

Among female cancers, cervical cancer has the second highest occurrence worldwide with an incidence in 2002 of 493,000 women (20% in developed countries and 80% in developing countries), with 274,000 estimated deaths [1]. High-risk Human Papilloma Virus genotypes are the most important risk factors for development of cervical cancer after infection of cervical epithelial cells [2].

Non-invasive cervical intraepithelial neoplasias (CIN) precede the development of invasive cancer and is much more frequent as the estimated risk for progression of a CIN2-3 lesion is less than 10%, furthermore the progression from CIN to (micro)invasive cancer can take 10-25 years [3]. Three grades are used by The World Health Organization to distinguish the degree of epithelial abnormality (CIN1, CIN2 and CIN3). These grades are associated with increasing risks for invasive cancer development, but CIN grades are not static events. A CIN lesion is a dynamic process that can progress to cancer, persist as the same CIN grade but also regress [4]. If left untreated, 5-30% of all histologically confirmed CIN2-3 lesions will over time develop invasive cancer [5]. Consequently all punch-biopsy confirmed CIN2-3 lesions are usually treated with diathermic cone excision [6]. This is a relatively aggressive therapy because up to 40% of CIN2-3 lesions will regress spontaneously without cone excision [7]. Cone excision may induce side effects, including cervical insufficiency, which is a serious late complication [8,9]. This may require hospitalization and immobilization of women with later pregnancy, from 16 weeks gestation. As the age of becoming pregnant rises, and the median detection age of CIN2-3 is 29 years only, the clinical importance of cervical insufficiency as a side effect increases. It is therefore of paramount importance to identify CIN2-3 lesions which could safely be treated with less aggressive therapy than cone excision, and find new diagnostic and prognostic predictive methods that can predict those CIN2-3 lesions that will regress spontaneously.

The distinction of a CIN2 lesion from a CIN3 lesion can be challenging. In addition, CIN2 lesions regress to a higher degree than CIN3 lesions, so an improvement in the diagnostic accuracy of high grade CIN's can potentially reduce the number of over treated patients [4].

Functional biomarkers like Ki67, pRb, p53 and cytokeratin 13/14 have proven to be helpful in predicting regression or not [4]. The type of immune-reactive cells in the microenvironment of a CIN lesion is also predictive for regression. One of the challenges is that the local immune response induced by the HPV-infection must be detected in formalin-fixed paraffin embedded (FFPE) tissue [10], which is used worldwide for the histopathological diagnosis of cervical lesions. The FFPE procedure virtually eliminates the availability of water-soluble proteins which could have diagnostic and prognostic value. Aggregated information provided by such biomarkers exceeds the value of the grading system. Establishment of new biomarkers to support prediction of regression or not may result in even more accurate CIN treatment [11].

Several studies have been performed using different sample collection and analysis technologies for different samples such as cervicovaginal washings [12,13], cervical mucus [14] and cells supernatant from cytobrush collection [15]. A protein collection method for small punch biopsy samples that could represent not only the cellular response but also water soluble proteins from the cervical neoplasia microenvironment may further help to define the biological dynamic behavior of CIN lesions. We have previously shown that a panel of 3 peaks from SELDI-TOF protein profiles can be used to differentiate normal tissue from CIN tissue samples [16] utilizing a method resembling the one published by Celis et al [17]. However, SELDI-TOF has certain shortcomings, and the aim of the present pilot study therefore was to compare CIN2 and CIN3 samples from this protein-saving biopsy processing method using shotgun proteomics [18]. We utilized nanoflow liquid chromatography coupled to a LTQ-Orbitrap mass spectrometer to identify proteins from the biopsies or the microenvironment to the neoplasia within the biopsies. At the same time, the tissue was preserved for conventional microscopic and immunohistochemical studies.

## Methods

### Study population

This study is a sub-project from a larger prospective observational study, approved by the Regional Medical Ethics Committee of Helse Vest, Norway, the Norwegian Data Inspection, and the Health Directorate of Norway, #33.06, #17185 and #07/330. In this observational study, healthy women aged 25-40 years, with cytologically abnormal smears were followed by cervical biopsy and later cone excision. In total, 254 patients with first time onset of CIN have been included from January 2007 to December 2008. The interval between punch biopsy and cone excision was standardized at median 113 days (range: 100-126). Ninety-five percent of the patients had an interval between 87 and 139 days. All patients included in this study were treated according to the national Norwegian population screening quality guidelines [19]. Of the 254 patients with cervical punch biopsy samples, a random subset of 10 diagnosed as CIN2 and 10 diagnosed as CIN3 were selected for the current pilot study. The samples were selected so that the whole sampling period was covered and the protein concentration was as close to the average for the whole data set as possible. The age and punch-cone excision interval of these 20 patients was not different from the whole group.

### Sample collection

After colposcopy, punch biopsies and endocervical curettage were taken from the transformation zone and eventually premalignant mucosal changes, and stored in 4% buffered formaldehyde, 1-2 additional biopsies were immediately placed in polystyrene tubes (Sarsted, Nurmbrecht, Germany) containing 5 ml RPMI 1640 (Gibco, Carlsbad, USA) tissue culture medium and kept there for 24 hours at 4°C. After this period, the supernatants were collected, split into aliquots of 500 μl and stored at -80°C until analysis.

### Pathology

As described before [16], after 24 hours incubation in RPMI1640 medium the biopsies were routinely fixed in buffered 4% formaldehyde, embedded in paraffin, cut at 4 μm, and stained with hematoxylin, eosin and safran (HES) for routine histological examination. P16 and MIB-1 immunohistochemical (IHC) stainings were used to confirm the diagnosis. All HES and IHC sections of the 254 biopsies were reviewed by two independent pathologists also using the p16 and MIB-1 immunohistochemical information, but who otherwise were blinded to the original routine clinical findings, histopathological diagnosis and follow-up. In case of discrepancies the cases were re-reviewed and diagnosed on a double-head microscope by two pathologists (EG, JB) and a consensus diagnosis was always obtained. The cases used for this study were diagnosed as follows: CIN2 (n = 10) and CIN3 (n = 10).

### Immunohistochemistry

Paraffin sections were mounted onto Superfrost Plus slides (Menzel, Braunschweig, Germany) and dried overnight at 37°C followed by 1 h at 60°C. Sections were deparaffinized in xylene and rehydrated in decreasing concentrations of alcohol. Antigen was retrieved with a highly stabilized retrieval system (ImmunoPrep, Instrumec, Oslo, Norway) using 10 mM TRIS/1 mM EDTA (pH 9.0) as the retrieval buffer. Sections were heated for 3 min at 110°C followed by 10 min at 95°C and cooled to 20°C.

Mouse monoclonal Cytokeratin 2e clone Ks2.342.7.1 (Abcam, Cambridge, UK,) were used. The primary antibody was used at a 1:25 dilution in Dako antibody diluent (S0809). The EnVisionTMFlex+ detection system (Dako, Glostrup, Denmark, K8002) were used for visualization. Sections were incubated for 5 min. with peroxydase-blocking reagent (SM801), 30 min. with the primary antibody, 20 min. with the EnVision™ FLEX+ Mouse Linker (SM804), 20 min. with the EnVision™ FLEX/HRP Detection Reagent (SM802), 10 min. with EnVision™ FLEX DAB+ Chromogen (DM827)/EnVision™ FLEX Substrate Buffer (SM803) mix and 5 min. with EnVision™ FLEX Hematoxylin (K8008). The slides were dehydrated and mounted. Immunohistochemical stainings were performed using a Dako Autostainer Link 48 instrument and EnVision™ FLEX Wash Buffer (DM831).

### Immunoaffinity depletion

The preparation of the immunoaffinity column is described in Additional file 1. To deplete samples of the 7 high-abundance proteins, 100 μl of RPMI supernatant was diluted with 100 μl tris-buffered saline (TBS, 0.1 M TRIS-base containing 0.1 M NaCl, pH 8.0), and the solution was injected into a TBS solution with a flow of 0.2 ml/min. The non-retained proteins were trapped on a 4 mm × 2.0 mm id. C_18 _Security Guard Cartridge with 300Å pore size (Phenomenex, Torrance, CA, USA), and were eluted by backflushing the security guard cartridge with ethanol at a flow of 0.3 ml/min. The affinity column was washed using 0.1 M glycine at pH 2.5 with a flow of 1.2 ml/min. Both columns were re-equilibrated with TBS at a flow of 0.2 ml/min for 5 minutes. The pH adjustments were done using 6 M HCl.

### Protein digestion and sample cleanup

After evaporating the ethanol-phase containing the non-retained protein fraction using vacuum-centrifugation (Eppendorf Concentrator 5301, VWR, Norway), 100 μl 50 mM ammonium-bicarbonate pH 8 was added to the samples. 1 μl of 1 M dithiothreitol (DTT) was added to reduce the proteins. 5 μl of 1 M iodoacetamide (IAA) was then added to alkylate the proteins followed by 5 μl of DTT to stop the alkylation process. For each of these steps, 45 minute incubation time was used. One μg trypsin (Promega) was added, and the samples were kept at 37°C for 18 hours. After trypsination, the samples were purified and concentrated using a C_18 _ZipTip (Millipore, Norway) procedure. The ZipTips were conditioned by aspiring 30 μl acetonitrile five times and equilibrated with pulling 30 μl 0.1% formic acid (FA) in MilliQ water five times through the stationary phase. Approximately 10 μl of the 0.1% FA solution was left above the stationary phase to avoid drying it. Each sample solution was applied on top of the stationary phase using a pipette, and then pushed through the tip using air pressure from the pipette plunger. More sample solution was added when approximately 20 μl of the liquid remained so that the whole volumes of the samples were pushed slowly through the ZipTip. Washing was done by aspiring 30 μl of 0.1% FA five times. Elution of the peptides was done in a total volume of 30 μl of 80:20 (v/v) acetonitrile:MilliQ water by aspiring 10 μl of this solution 10 times through the stationary phase. The organic phase was then evaporated using vacuum-centrifugation and to the residual solution, 20 μl 0.1% FA was added prior to the LC-MS/MS analysis.

### LC-MS/MS analysis

A Dionex Ultimate 3000 nanoflow HPLC equipped with a 300 μm i.d × 0.5 cm length Acclaim PepMap 100 C_18 _trap column and a 75 μm id. × 15 cm Acclaim PepMap 100 C_18 _analytical column (Dionex) were used with a LTQ-Orbitrap hybrid mass spectrometer (Thermo Scientific). 5 μl of the tryptic digests were injected onto the trap column using 0.1% formic acid (FA, VWR) in MilliQ-water (Elga) at a flow of 2 μl/min. The separation was done using a gradient from 2.5% to 64% acetonitrile in 0.1% FA over 180 minutes using 300 nl/min flow. A 10 minute post-injection delay and a 20 minute column re-equilibration time were used. The electrospray interface was a PicoTip emitter (SilicaTip, New Objective) with a 10 μm tip without coating. The electrospray voltage was set to 1 kV. No sheath gas was used. The mass spectrometer was used in positive mode. Full scans were performed in the Orbitrap using the m/z range from 200 to 2000. Data dependent MS/MS scans were performed in the LTQ for the five most abundance masses with z ≥2 and intensity higher than 10000 counts. Dynamic exclusion for 3 minutes after fragmentation of a given m/z value four times was used. Collision induced dissociation (CID) was used with a collision energy of 35%, activation Q setting of 0.400 and 30 ms activation time for MS. Calibration of the mass spectrometer was done weekly using the calibration solution recommended by Thermo Scientific.

### Data analysis

The raw data files were analyzed using the Proteome Discoverer 1.0 (Thermo Scientific) with the Sequest algorithm to search against the Homo Sapiens (Tax.id: 9606) database at NCBI (531420 sequences) with trypsin as digestion enzyme allowing for 2 missed cleavages. All files were also searched against the Human Papilloma Virus database (Tax.id: 10566) at NCBI (1615 sequences). Precursor ion tolerance was set to 10 ppm, and fragment ion mass tolerance to 0.8 Da. Oxidation (M) was set as a dynamic modification and carbamidomethyl (C) was set as a static modification due to the use of DTT and IAA. A high significance peptide confidence filter was set in Proteome Discoverer (PD) from Thermo, which means that peptide identifications are filtered based on the following combination of charge and Xcorr factor: 1.9 (z = 2), 2.3 (z = 3) and 2.6 (z≥4). The InforSense plugin to Proteome Discoverer was used to collect gene ontology (GO) data. Information of other relevant biological processes for each protein was obtained from the Amigo database (http://amigo.geneontology.org).

**Figure 1:**
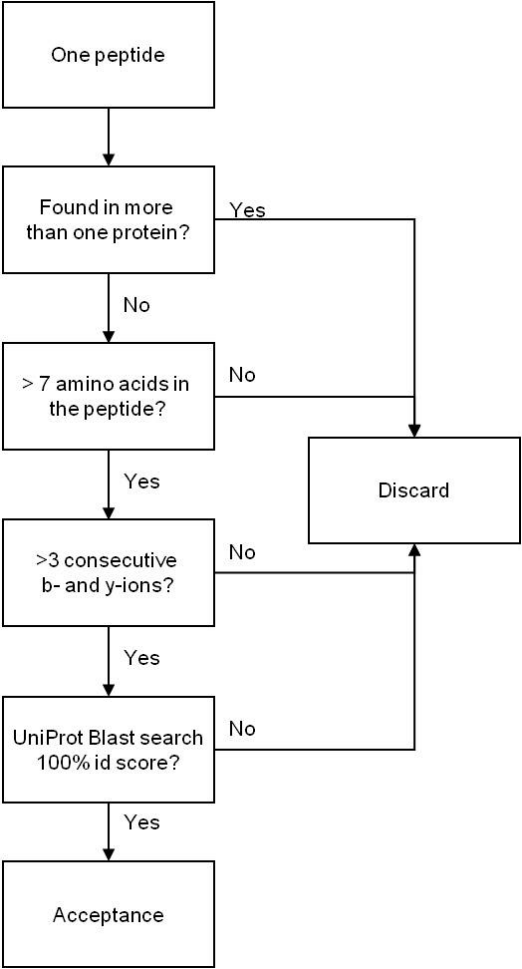
**Flow chart for the acceptance process for protein identification using one peptide**.

To enable identification of proteins with low molecular weights that can result in one or only a few tryptic peptides, protein identifications were accepted using one peptide under certain conditions: The sequest Xcorr factor with regards to charge had to be fulfilled according to the high significance criteria in PD. The peptide had to contain at least 7 amino acids, and at least three consecutive b- and y-ions had to be present in the spectra [20], and it should be found minimum three times in the same sample. In addition, for proteins with only one identified peptide sequence, the peptide sequence were submitted to a BLAST search against the Uniprot Homo Sapiens database (http://www.uniprot.org) to confirm that the identification matched the NCBI identification. The identification process is described in figure 1. For proteins listed as Unnamed in the NCBI database, the ID mapping tool at UniProt was used to see if the protein was listed with a more descriptive annotation in this database.

Spectral count (SPC) results for the identified peptides were obtained and used for normalization (equation 1) after increasing all numbers with 1 to avoid zeros.

The normalized SPC results were then imported into SPSS (v17, SPSS, Oslo, Norway) for statistical calculations using descriptive statistics and for developing a binary logistic regression model using the Forward Wald method. A Classification and Regression Tree analysis was done using CART (Salford, San Diego, CA, USA).

## Results

The median age of the patients at the time that the biopsies were obtained was 29.7 years (range 25-40), and the mean protein concentration of the selected samples, measured by Bradford, was 0.81 mg/ml (range 0.55 - 1.14). The samples were subjected to depletion of 7 high abundance proteins followed by tryptic digestion and uni-dimensional LC-MS/MS analysis. Using the high-significance peptide confidence filter in Proteome Discoverer, peptides from a total of 193 protein entries were identified. Applying the identification criteria for proteins with only one peptide, a total of 114 protein identifications were included (table 1). Thirty six of these were identified with two or more unique peptides and the rest with only one unique peptide. Peptides from Human Papilloma Virus proteins were detected in all samples, but not with high enough confidence to give acceptable protein identifications. Albumin, haemoglobins and immunoglobulins were excluded from the protein list in table S1 in Additional file 2 despite that they fulfilled the protein identification criteria.

**Table 1:** Classification table showing the results from the binary logistic regression model

			Predicted		
			Group		
	Observed		CIN2	CIN3	Percentage Correct
Step 1	Group	CIN2	16	4	80.0
		CIN3	0	20	100.0
	Overall Percentage				90.0

A cut-value of 0.250 was used.

**Figure 2:**
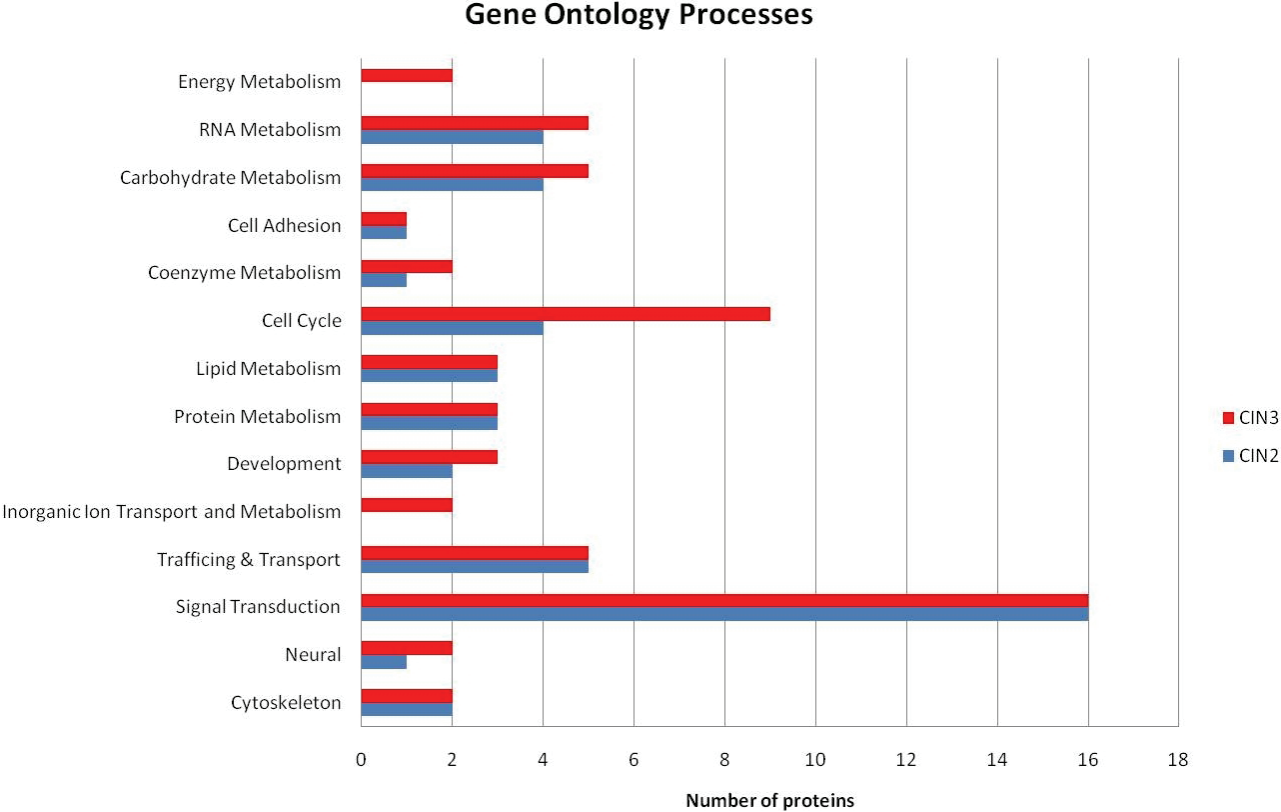
**A bar diagram showing the number of proteins found in the CIN2 and CIN3 groups covering different gene ontology processes**.

Figure 2 show a gene ontology (GO) bar diagram for the biological processes covered by the proteins in the CIN2 and CIN3 groups identified by Proteome Discoverer.

The InforSense module could assign GO processes to 46 (53%) and 60 (53%) of the identified proteins in the CIN2 and CIN3 group, respectively. Among these 46 and 60 proteins with assigned GO process, the most frequent occurring processes are signal transduction with 16 proteins in both groups, which is 35% of the proteins in the CIN2 group and 27% of the proteins in the CIN3 group. Proteins related to cell cycle were represented by 4 proteins in the CIN2 group (9%) and 9 in the CIN3 group (15%). In total, 15 (33%) and 20 (33%) of the proteins with a GO annotation are involved in metabolic processes in the CIN2 and CIN3 groups respectively. In the CIN3 group, proteins involved in energy metabolism (2) and inorganic ion transport and metabolism (2) were identified as well.

**Figure 3:**
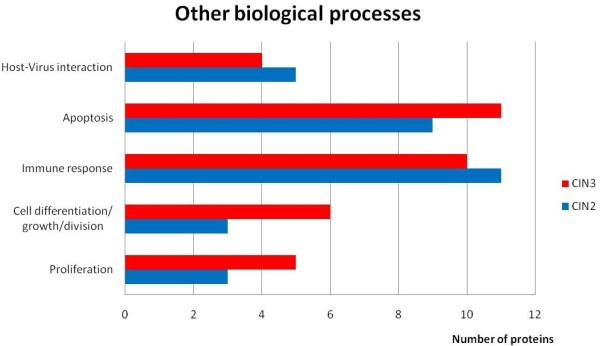
**A bar diagram showing the number of proteins covering other biological processes related to cell development and host-virus interactions**.

Several of the identified proteins are related to other biological processes than is shown in figure 2, and was obtained from Amigo (figure 3). Proteins involved in immune responses are heat shock protein beta-1, apolipoprotein A-I, alpha-1-acid glycoprotein 1, dermcidin, macrophage migration inhibitory factor and cystatin-C. Other proteins have been shown to be involved in host-virus interactions or virus response mechanisms, for example high motility group protein AT-hook protein 1, hemopexin, apolipoprotein A-II, keratins 8 and 19, Heat shock protein beta-1 and vimentin.

The classification results obtained by developing a binary logistic regression model based on the normalized spectral counts are shown in table 1.

**Figure 4:**
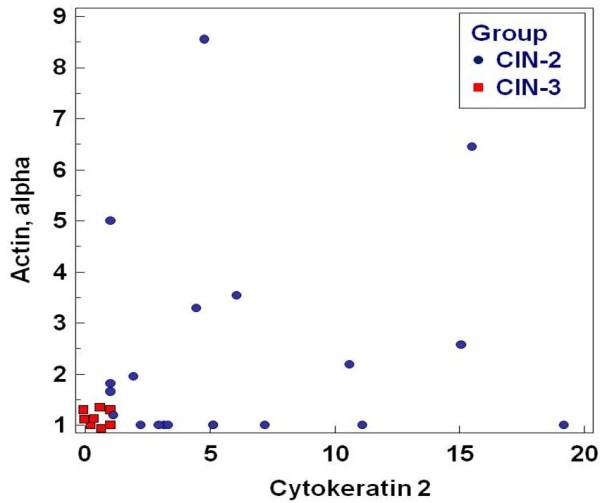
**Scatterplot showing the discrimination between CIN2 and CIN3 using cytokeratin 2 and actin alpha**.

A 90% overall correct classification of CIN2 and CIN3 was obtained with Cytokeratin 2 as the significant discriminatory factor. The CART analysis resulted in a 97% correct classification using Cytokeratin 2 and actin alpha as discriminating factors. Figure 4 shows a scatterplot using these two proteins.

**Figure 5:**
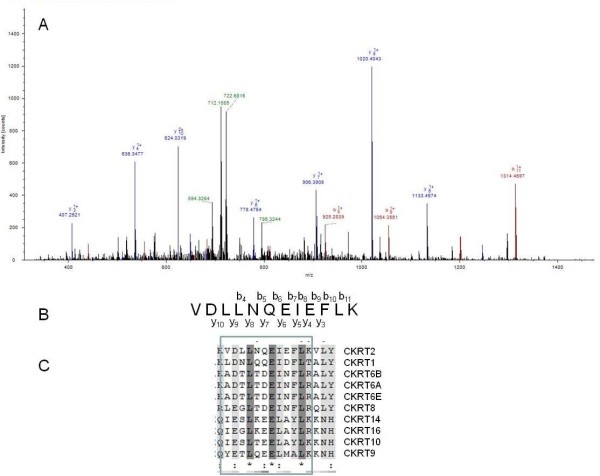
**MS2 spectrum of the peptide with the sequence VDLLNQEIEFLK (A), the sequence with the y- and b-series shown (B) and the part of the sequence containing this peptide shown in a multialignment of all identified cytokeratins**.

Cytokeratin 2 was identified with five peptides. An MS2 spectrum for one of these peptides is shown in figure 5 panel A. Panel B in the figure show the y- and b-series with the peptide sequence, and panel C a multi-alignment of this part of the protein sequence with all the other cytokeratins identified in this study. The multi-alignment shows that this peptide makes Cytokeratin 2 (CK-2) different from the other cytokeratins identified.

**Figure 6:**
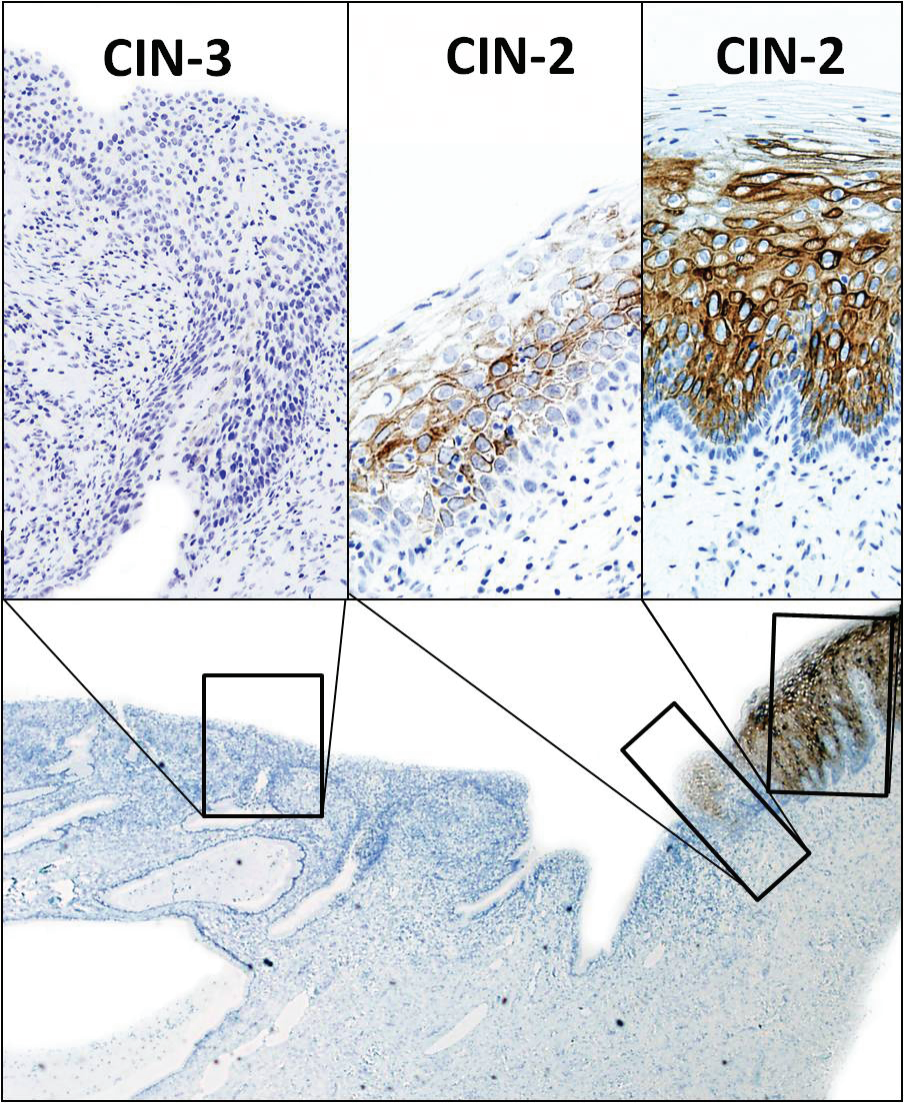
**Results from a preliminary study showing that CIN-3 lesions are completely negative, while CIN-2 lesions reveal different expressions of Cytokeratin 2 (CK-2)**. Note that the basal cells are negative for CK-2.

In a small preliminary immunohistochemical study, we found that normal epithelial cells in the lower half are CK-2 positive (but not the basal cells). CIN-3 lesions are negative. CIN-2 lesions show CK-2 expression in the low and middle part of the epithelium, but the cells are not as strongly positive as the normal epithelium, and in a patchy manner (figure 6).

## Discussion

This study shows that CIN biopsies shed a complex mixture of proteins into a cell culture medium for 24 hours at 4°C. Supernatants from 20 patient samples were analyzed using a bottom-up shotgun proteomics approach [21] in which the proteins were digested into smaller peptides using trypsin. The peptide mixture was then analyzed using uni-dimensional LC-MS/MS. Despite the depletion of seven high abundance proteins including immunoglobulins and albumin, peptides from these proteins were detected, while transferrin was not found at all after depletion. In addition, not unexpectedly haemoglobins constitute a relatively large part of the identifications (cervical tissues with CIN2-3 are usually richly vascularised) and should be included in future depletion work. The protein mixtures still are of such complexity after depletion that it would be advantageous to do further fractionations by for example using two-dimensional separations like 2D gels or MudPit [22]. Selective enrichments of for example phosphorylated kinases that are important components in regulation of the cell cycle [23] would reduce the complexity of the samples while at the same time enrich interesting proteins. The glycosylation pattern of proteins is another interesting topic [24,25] that could be further elucidated using this sample set.

The gene ontology bar diagram in figure 2 shows that 33% of the proteins identified are annotated to metabolic processes, 35% to signal transduction in CIN2 and 27% in CIN3, 9% annotated to cell cycle processes in CIN2 and 15% in CIN3, and annotated to trafficking/transport. In agreement with our results, Panicker et al [26] reported that 33% of the identified proteins in cervical mucus samples were related to metabolism, while Dasari et al [27] reported 32% after analysis of cervical-vaginal fluid. The fraction of the proteins involved in signal transduction processes (35 and 27%) in this study is much higher than the results from the studies by Panicker et al [26] and Dasari et al [27], who reported 1% and 3%, respectively. This may be due to the higher efficiency of the exudation process of a biopsy (which in principle has a much larger direct stroma contact surface with the RPMI than intact cervical epithelium has with cervical mucus or vaginal fluid) and the long incubation time we have used (24 hours).

Several of the proteins found in this study have been reported by others in cervical mucus samples [26], and also in plasma samples from patients with CIN [28] or in cervical tissue samples [29-32]. Vimentin was found down-regulated in vaginal and cervical carcinoma compared to normal tissue [31,32]. Actin, transthyretin, lamin a/c, fibrinogen and apolipoprotein A-I are all proteins identified in one or more of the mentioned studies, and several of these proteins have been connected to cancer or used as cancer markers [33-35]. Transthyretin has been used as a biomarker for nutritional status and inflammation, but post-translational modified forms have also been reported as part of a biomarker panel for early detection of ovarian cancer [36,37]. Transthyretin was found in 6 of the 10 CIN3 samples and none of the CIN2 samples. Alpha-1-acid glycoprotein (AGP) is known to increase during acute-phase response, [38], and has also been identified in other studies [26,27] using cervical vaginal fluid or mucus. The heat shock proteins are involved in a range of cell processes. They are induced under stress conditions and known to be over-expressed in human cancers. Some of them are used as biomarkers for carcinogenesis and some as signals for aggressiveness of some cancers [39]. Two studies [30,31] found the level of heat shock protein 1 (Hsp27) to be up-regulated in carcinoma samples. However, another study discovered a decline [29] and related this to the presence of HPV oncoproteins with a negative effect on the ability of lesions to undergo terminal differentiation. We found no significant difference between CIN2 and CIN3 samples with regards to Hsp27 based on spectral count comparison.

A conserved 7 amino acid sequence found in lipocalins, including the lipocalin-type prostaglandin D synthase (L-PGDS) was identified in 17 of the 20 samples in this study and was recently demonstrated to modulate cell survival [40]. Lipocalins are normally present at low concentrations, but the expression can increase due to physiological conditions [40]. The tumor suppressor p53 has been found to suppress the expression of L-PGDS [41]. A decrease in the p53 level, as found in most CIN2-3 lesions [4], combined with a local inflammation, might explain the frequent occurrence of L-PGDS in the CIN samples.

Proteins from the intermediate filament (IF) protein family is highly represented among the proteins identified in both the CIN2 and CIN3 group. The cytokeratins belong to the type I and II of the IF's, desmin and vimentin belong to the type III and lamin to type V. This group of proteins have been widely used as markers of different cancers [42].

Special attention should be paid to the late epithelial cell differentiation marker Cytokeratin 2 (CK-2), which was found to have highest discriminatory power between CIN2 and CIN3, with 90% correct classification. The protein was identified using 5 different peptides (2 unique), and only in samples from the CIN2 group (in 8 of the 10 samples).

This protein is expressed late in the differentiation process in the uppermost epidermal layers of the normal skin [43], and has normally been associated with the skin disorder ichthyosis bullosa of Siemens [44]. The protein was characterized by Collin et al [45,46], and was found overexpressed in patients with head and neck squamous cell cancer [47]. The fact that CK-2 is identified in CIN-2 lesions only indicates that the epithelial cells have a greater tendency to high-end differentiation than CIN3 lesions, which is biologically well understandable. To our knowledge, this is the first report of Cytokeratin 2 associated with differences between CIN2 and CIN3, or with other neoplasia [48].

Although the number of samples is small, the proteomics results have been confirmed by immunohistochemical evaluation, when performed, and further validation is in progress.

A differentiation between a CIN2 and CIN3 diagnosis has at the moment no consequence for patients with regards to follow-up or treatment as all these patients in principle will undergo surgical cone excision. However, the fact that there are differences at the protein level amongst high grade CIN lesions with microscopically different epithelial appearances, as detectable by expert pathologists supported by p16 and MIB-1 immunohistochemistry, make clear that these differences are real and may have biological impact. These additional novel markers could help pathologists in differentiating CIN2 and CIN3. This may be especially of interest as high grade CIN 3 lesions have a lower likelihood to regress spontaneously and a higher probability to progress to invasive cancer, than CIN 2 lesions [3]. The fact that this well-known knowledge currently is not used in therapeutic decision making, is due to the lack of reproducibility amongst pathologists when classifying CIN 2 and CIN3 lesion with conventional Hematoxyllin and Eosin stained sections alone. However, with the advent of new molecular immunohistochemical biomarkers, and possibly also proteins and peptides isolated with the water soluble strategy as described in the current study, the distinction could become of clinical interest.

## Conclusions

The current study led to identify 114 proteins, including several ones that had been previously identified by others [26,27,29-32]. The late epithelial cell differentiation marker Cytokeratin 2 was found to have the highest discriminatory power between CIN2 and CIN3, with 90% correct classification, thus suggesting a role for Cytokeratin 2 as a grading marker in CIN. As a whole, the study highlights the informative potential in terms of either biologic knowledge or diagnostic refinement that is inherent in a proteomic analysis of cancer supernatants.

## Disclosure/Conflict of interest

The authors declare that they have no competing interests.

## Authors' contributions

KEU. JB, EJ and AH designed the study. KEU performed the sample preparation, analysis and bioinformatics. KEU and JB did the statistical calculations. CB prepared the depletion column and depletion methodology. ACM, EG, EJ, IS and BD contributed to the study design. All authors have been critically involved in the manuscript drafting, and all authors have read and approved the final manuscript.

## Supplementary Material

Additional file 1 **Additional file 1_Preparation of depletion column.doc**. A description of how the depletion column was prepared. Click here for file

Additional file 2 **Additional file 2_TableS1_List of identified proteins**. A table listing the identified proteins with NCBI accession number, description, molecular weight, #AA, #peptides and % coverage. Click here for file
