# Supplementary material for: Discrimination of grade 2 and 3 cervical intraepithelial neoplasia by means of analysis of water soluble proteins recovered from cervical biopsies
Source: Proteome Sci. 2011 Jun 28;9:36. doi: 10.1186/1477-5956-9-36 (PMC3142202; doi:10.1186/1477-5956-9-36)
Supplement: Additional file 1 — _Preparation of depletion column.doc. A description of how the depletion column was prepared. [file 1477-5956-9-36-S1.DOC]

SUPPLEMENTARY MATERIAL.

***Preparation and use of an immunoaffinity extraction column to deplete high abundance proteins***

The material in the immunoaffinity extraction column was prepared by coupling antibodies onto Toyopearl AF-Tresyl-650M (Tosoh Bioscience, Tokyo, Japan). This is a tresyl-activated polymeric affinity resin with 65 µm particle size and 1000 Å pore size, to which proteins are coupled by their primary amine groups (lysine). Polyclonal antibodies were obtained from Roche (Basel, Switzerland), and were originally intended for use in immunoturbidimetric analytical methods. Coupling reactions were done in three batches, each with different types of antibodies (details shown in Table 1). To avoid coupling of TRIS to the resin, the TRIS-buffers in the original antibody solutions were changed to saline water by dialysis. Each batch of antibody was separately transferred to 50 cm long 12 kDa Spectrapor dialyzer tubings (Thomas Scientific, Philadelphia, USA) sealed by tight knots. A total of three tubings were contained in a plastic can of 5 L volume, filled with RO water, and kept at 4 °C. After 24 h, the water was changed to saline water comprising of 0.1 % (w/v) NaCl, then after another 24 h back to water, and finally after 24 h back to saline water again. The three antibody solutions were then transferred to separate 20 ml polystyrene tubes with screw corks (Sterilin, UK), followed by addition of tresyl-activated resin, 0.58 g NaCl (0.5 M), and 0.17 g Na_2_HCO_3_ (0.1 M) to give pH 8.3 for the coupling reaction. The tubes were rotated slowly for 5 h at room temperature, followed by addition of 1.7 g NaCl, and rotated for another 1 h at room temperature. After sedimentation of the resin, the solutions were discarded and the resin washed three times with 15 ml PBS. The tubes were then filled with TRIS-buffer (0.1M, pH 8) for deactivation of remaining active sites and kept at 4°C overnight. All three batches were finally mixed carefully together into one polystyrene tube and kept in TRIS-buffer (0.1M, pH 8) with 0.01 % (w/v) Na-azide added as antimicrobial agent.

Approximately 1.3 ml slurry of the immunoaffinity adsorbent material was transferred to an empty 100 mm long and 4.0 mm in diameter (id) PEEK column (part no.JR-68182NF, VICI, Houston, USA) using 7.9 mm id. frits in both ends (part no. JR1124-5P, VICI). The packing of the column bed was facilitated by drawing buffer out of the end filter using a 20 ml plastic syringe.

Table 1 Antibody solutions and amount of activated resin in each batch of affinity material produced.

| Batch number | Antibody | Manufacturing animal | Approximate concentrations | Roche catalogue number | Volume used in the batch (ml) | Amount of activated resin (g) |
| --- | --- | --- | --- | --- | --- | --- |
| 1 | Anti-human albumin | Sheep | 15 g/l | 04469658 | 15 | 2.0 |
| 2 | Anti-human transferrin | Rabbit | 2 g/l | 04580524 | 8 | 1.0 |
|  | Anti-human α1-antitrypsin | Rabbit | 2 g/l | 11557599 | 4 |  |
|  | Anti-human haptoglobin | Rabbit | 1 g/l | 11557629 | 4 |  |
| 3 | Anti-human IgA | Goat | 10 mg/l | 03507297 | 3 | 0.70 |
|  | Anti-human IgM | Goat | 10 mg/l | 03507149 | 3 |  |
|  | Anti-human IgG | Goat | 10 mg/l | 03507408 | 10 |  |
