# Supplementary material for: Discrimination of grade 2 and 3 cervical intraepithelial neoplasia by means of analysis of water soluble proteins recovered from cervical biopsies
Source: Proteome Sci. 2011 Jun 28;9:36. doi: 10.1186/1477-5956-9-36 (PMC3142202; doi:10.1186/1477-5956-9-36)
Supplement: Additional file 2 — _TableS1_List of identified proteins. A table listing the identified proteins with NCBI accession number, description, molecular weight, #AA, #peptides and % coverage. [file 1477-5956-9-36-S2.DOC]

Table S1: Protein identifications.

| No. | GI Number | #AAs | MW [Da] | Protein description^a^ | ΣCoverage | # Peptides | pI (Theor.) |
| --- | --- | --- | --- | --- | --- | --- | --- |
| 1 | 10281111 | 444 | 50333 | NADPH oxidase subunit gp91-3 | 4.05 | 1 | 8.28 |
| 2 | 115528983 | 113 | 12481 | AOF1 protein | 15.93 | 1 | 4.86 |
| 3 | 118764045 | 1691 | 188664 | ADAMTS-like 3 | 0.65 | 1 | 8.21 |
| 4 | 119576082 | 73 | 8489 | ATP synthase mitochondrial F1 complex assembly factor 2 | 24.66 | 1 | 6.62 |
| 5 | 119581148 | 529 | 57526 | Keratin 9 | 35.16 | 12 | 5.14 |
| 6 | 119581152 | 261 | 29961 | Keratin 14 | 6.90 | 2 | 5.09 |
| 7 | 119582219 | 1064 | 120637 | Serine peptidase inhibitor, Kazal type 5 | 0.94 | 1 | 8.45 |
| 8 | 119587706 | 91 | 10166 | Transgelin | 13.19 | 1 | 8.87 |
| 9 | 119598796 | 79 | 9477 | NADH dehydrogenase (ubiquinone) 1 beta subcomplex | 18.99 | 1 | 9.62 |
| 10 | 119599670 | 213 | 22474 | H1 histone family, member X | 5.63 | 1 | 10.76 |
| 11 | 119600188 | 110 | 12963 | Ribosomal protein L24 | 11.82 | 1 | 11.26 |
| 12 | 119602169 | 3509 | 403057 | Dynein, cytoplasmic 1 | 0.43 | 1 | 6.01 |
| 13 | 119603728 | 102 | 11255 | hCG2013819 (Protein S100-A11) | 15.69 | 1 | 6.56 |
| 14 | 119606009 | 525 | 56446 | Heparan sulfate (glucosamine) 3-O-sulfotransferase 6 | 3.43 | 1 | 10.8 |
| 15 | 119608171 | 617 | 68944 | Golgi autoantigen, golgin subfamily a | 1.78 | 1 |  |
| 16 | 119612128 | 217 | 24714 | dpy-19-like 4 (C. elegans) | 7.37 | 1 | 8.41 |
| 17 | 119612724 | 272 | 30270 | Actin, alpha, cardiac muscle | 18.38 | 3 | 5.23 |
| 18 | 119614748 | 1479 | 166549 | Mannose receptor, C type 2 | 0.95 | 1 | 5.54 |
| 19 | 119617032 | 563 | 59874 | Keratin 6B | 11.72 | 7 | 8.09 |
| 20 | 119619399 | 294 | 31511 | hCG1814593 | 6.12 | 1 |  |
| 21 | 119624177 | 104 | 11123 | High mobility group AT-hook 1 | 24.04 | 1 | 10.31 |
| 22 | 119632213 | 237 | 26576 | RAB33A, member RAS oncogene family | 8.44 | 1 | 8.07 |
| 23 | 123238660 | 300 | 31702 | Ribosome binding protein 1 homolog | 4.33 | 1 | 9.89 |
| 24 | 13279239 | 272 | 29864 | ENO1 protein | 10.66 | 2 | 5.62 |
| 25 | 1335098 | 439 | 49264 | Unnamed protein product (Hemopexin) | 7.97 | 2 | 6.55 |
| 26 | 134105414 | 77 | 8702 | Chain L, Apolipoprotein A-Ii | 96.10 | 6 | 5.05 |
| 27 | 13431717 | 1938 | 223540 | Myosin-13 | 0.83 | 1 | 5.56 |
| 28 | 13529281 | 254 | 28612 | HPX protein | 4.33 | 1 | 6.45 |
| 29 | 1419564 | 279 | 30759 | Cytokeratin (Type II Cytoskeletal 8) | 16.85 | 5 | 5.52 |
| 30 | 159162253 | 60 | 6919 | Chain A, An Active Mini-Proinsulin, M2pi | 71.67 | 2 | 6.9 |
| 31 | 159162826 | 59 | 6576 | Chain 2, Intestinal Trefoil Factor (Tff3) | 30.51 | 2 | 5.13 |
| 32 | 159163486 | 155 | 17209 | Chain A, Transgelin-2 | 7.74 | 1 | 8.45 |
| 33 | 159164754 | 107 | 12623 | Chain A, Mrf2-Dna Complex | 14.95 | 1 | 8.89 |
| 34 | 15928913 | 191 | 21129 | Unknown (protein for IMAGE:3906970) (Heat Shock Protein beta 1) | 22.51 | 3 | 5.98 |
| 35 | 160332335 | 5890 | 628699 | Neuroblast differentiation-associated protein AHNAK; AltName: Full=Desmoyokin | 23.28 | 51 | 5.8 |
| 36 | 16552261 | 410 | 47459 | Unnamed protein product (Vimentin) | 49.02 | 20 | 5.06 |
| 37 | 167013484 | 80 | 8912 | Chain D, Saposin D (Triclinic) | 13.75 | 1 | 5.06 |
| 38 | 168985213 | 349 | 37829 | GTP binding protein 1 | 4.01 | 1 | 10.89 |
| 39 | 171702853 | 92 | 10339 | Ferritin light polypeptide | 33.70 | 2 | 5.62 |
| 40 | 181965 | 227 | 24181 | Elongation factor 1 alpha | 22.47 | 3 | 9.61 |
| 41 | 182354 | 135 | 15155 | Fatty acid binding protein homologue | 10.37 | 1 | 6.82 |
| 42 | 183758 | 47 | 5010 | Histone H2A | 19.15 | 1 | 11.05 |
| 43 | 186972736 | 79 | 8759 | Chain A, Apolipoprotein C-Iii | 21.52 | 1 | 4.72 |
| 44 | 187950363 | 458 | 49555 | Keratin 13 | 3.06 | 2 | 4.91 |
| 45 | 1911723 | 224 | 25823 | Na+/H+ exchanger isoform NHE-3 | 11.61 | 1 | 9.98 |
| 46 | 193785614 | 127 | 14536 | Unnamed protein product (cDNA FLJ11717 fis, clone HEMBA1005241) | 14.96 | 1 | 6.14 |
| 47 | 193786857 | 603 | 68968 | Unnamed protein product (cDNA FLJ33218 fis, clone ASTRO2000381, highly similar to Huntingtin-interacting protein 1) | 2.99 | 1 | 6.23 |
| 48 | 194375299 | 333 | 37325 | Unnamed protein product (cDNA FLJ58286, highly similar to Actin, cytoplasmic 2) | 10.21 | 2 | 5.49 |
| 49 | 194377108 | 161 | 17039 | Unnamed protein product (Phosphoglycerate kinase) | 6.83 | 1 | 5.47 |
| 50 | 194377194 | 520 | 55765 | Unnamed protein product (cDNA FLJ51361, highly similar to Keratin, type II cytoskeletal 6A) | 17.69 | 6 | 6.12 |
| 51 | 194377570 | 188 | 21820 | Unnamed protein product (cDNA FLJ56690, highly similar to Tropomyosin beta chain) | 18.62 | 2 | 4.78 |
| 52 | 194379984 | 574 | 63952 | Unnamed protein product (cDNA FLJ56081, highly similar to Lamin-A/C) | 8.71 | 5 | 6.72 |
| 53 | 194382534 | 126 | 13563 | Unnamed protein product (cDNA FLJ50331, highly similar to F-box only protein 28) | 14.29 | 1 | 6.71 |
| 54 | 194388850 | 547 | 58643 | Unnamed protein product (cDNA FLJ54379, highly similar to Keratin, type II cytoskeletal 6E) | 18.10 | 7 | 7.58 |
| 55 | 194389168 | 612 | 65979 | Unnamed protein product (cDNA FLJ58491, highly similar to Homo sapiens hypoxia inducible factor 3, alpha subunit) | 2.78 | 1 | 6.81 |
| 56 | 194391072 | 352 | 39489 | Unnamed protein product (Vitamin D binding protein) | 21.88 | 6 | 5.22 |
| 57 | 195972866 | 584 | 58766 | Keratin, type I cytoskeletal 10 | 20.32 | 7 | 5.13 |
| 58 | 20138912 | 118 | 12887 | Myotrophin | 14.41 | 1 | 5.28 |
| 59 | 211939376 | 152 | 17015 | Chain L, Coagulation Factor Viia Mutant | 9.87 | 1 | 5.03 |
| 60 | 21358854 | 467 | 53216 | Mutant desmin | 15.42 | 8 | 7.73 |
| 61 | 21465525 | 223 | 24470 | Chain A, Prokallikrein 6 (Hk6) PROZYME PROPROTEASE M Proneurosin | 14.35 | 2 | 5.21 |
| 62 | 215415640 | 241 | 27779 | Unnamed protein product (Apolipoprotein A-I) | 37.34 | 8 | 5.56 |
| 63 | 215794600 | 192 | 22657 | Alpha 1 Acid Glycoprotein | 16.67 | 3 | 4.93 |
| 64 | 21754182 | 369 | 40889 | Unnamed protein product (Paralemmin-2) | 4.34 | 1 | 6.91 |
| 65 | 223132 | 66 | 7200 | Inhibitor,Kunitz type proteinase | 18.18 | 1 | 5.95 |
| 66 | 229002090 | 44 | 4849 | CD44 molecule | 27.27 | 1 | 5.04 |
| 67 | 237823923 | 562 | 60848 | Chain J, Fibrinogen | 11.21 | 5 | 5.99 |
| 68 | 238054406 | 644 | 65999 | Keratin, type II cytoskeletal 1 | 37.11 | 22 | 3.92 |
| 69 | 239938650 | 639 | 65393 | Keratin, type II cytoskeletal 2e | 9.39 | 5 | 5.79 |
| 70 | 24234699 | 400 | 44079 | Keratin, type I cytoskeletal 19 | 7.50 | 3 | 8.15 |
| 71 | 24430192 | 473 | 51236 | Keratin, type I cytoskeletal 16 | 14.80 | 6 | 8.07 |
| 72 | 2627129 | 609 | 0 | Polyubiquitin | 21.02 | 2 | 6.56 |
| 73 | 27477127 | 513 | 56616 | Keratin, type II cuticular Hb2 | 1.95 | 1 | 6.4 |
| 74 | 2809059 | 1324 | 136398 | Insulin receptor substrate-2 | 2.57 | 1 | 8.9 |
| 75 | 282403527 | 88 | 9665 | Chain A, Heterochromatin Protein 1-Binding Protein 74 Histone H1 Like Domain | 11.36 | 1 | 9.69 |
| 76 | 283806779 | 176 | 19711 | Chain B, Lipocalin-Type Prostaglandin D Synthase | 27.89 | 2 | 8.37 |
| 77 | 289526762 | 30 | 3428 | Insulin | 100.00 | 2 | 5.22 |
| 78 | 290559958 | 48 | 4816 | Chain A, Dermcidin-1l, A Human Antibiotic Peptide | 58.33 | 2 | 5.64 |
| 79 | 290789854 | 67 | 7725 | Chain A, Calcium-Calmodulin | 23.88 | 1 | 4.09 |
| 80 | 29649713 | 84 | 9161 | Protease serine 1 | 15.48 | 1 | 10.28 |
| 81 | 298508360 | 114 | 12337 | Chain C, Macrophage Migration Inhibitory Factor | 9.65 | 1 | 8.24 |
| 82 | 30023 | 765 | 69302 | Unnamed protein product (Collagen alpha-2 chain) | 2.35 | 1 | 10.05 |
| 83 | 3153859 | 84 | 9315 | Thioredoxin delta 3 | 13.10 | 1 | 5.8 |
| 84 | 339685 | 117 | 12835 | Transthyretin | 47.01 | 4 | 5.35 |
| 85 | 34596876 | 305 | 35093 | GPRA isoform F | 4.92 | 1 | 8.63 |
| 86 | 38197116 | 266 | 29985 | CAMKK2 protein | 4.14 | 1 | 6.25 |
| 87 | 386959 | 93 | 10828 | Migration inhibitory factor-related protein 8 (S100A8) | 36.56 | 2 | 6.51 |
| 88 | 39645240 | 705 | 78333 | HNRPU protein | 2.55 | 1 | 5.76 |
| 89 | 40041422 | 127 | 13370 | Unnamed protein product (Superoxide Dismutase Cu-Zn) | 44.09 | 3 | 5.7 |
| 90 | 40041430 | 81 | 0 | Unnamed protein product (Tubulin alpha-2) | 18.52 | 1 | 4.98 |
| 91 | 40046488 | 122 | 13954 | Unnamed protein product (Fibrinogen gamma-chain) | 18.85 | 1 | 5.37 |
| 92 | 417101 | 213 | 21352 | Histone H1.2 | 6.10 | 2 | 10.94 |
| 93 | 4704623 | 1246 | 132034 | APC2 protein | 1.36 | 1 | 9.08 |
| 94 | 473714 | 439 | 47377 | RNA helicase | 4.56 | 1 |  |
| 95 | 48735296 | 136 | 14535 | Signal recognition particle 14kDa | 21.32 | 1 | 10.05 |
| 96 | 50949605 | 738 | 80756 | Hypothetical protein | 2.17 | 1 | 4.78 |
| 97 | 54304028 | 86 | 9195 | Glyceraldehyde-3-phosphate dehydrogenase | 17.44 | 1 | 9.72 |
| 98 | 553778 | 87 | 10176 | T-cell receptor beta | 14.94 | 1 | 6.89 |
| 99 | 55669629 | 110 | 12320 | Chain H, N-Truncated Human Cystatin C | 18.18 | 2 | 8.75 |
| 100 | 55959888 | 97 | 10670 | Peroxiredoxin 1 | 11.34 | 1 | 8.27 |
| 101 | 56675572 | 218 | 0 | Unnamed protein product (Ribosomal protein L14) | 10.09 | 1 | 10.94 |
| 102 | 6014587 | 398 | 43783 | Mesothelin/megakaryocyte potentiating factor | 3.52 | 1 | 5.77 |
| 103 | 62087548 | 498 | 55683 | L-plastin variant | 3.41 | 1 | 5.21 |
| 104 | 62467428 | 987 | 113684 | Proteasome activator 200 kDa iii | 1.52 | 1 | 6.45 |
| 105 | 63021422 | 169 | 18128 | Small proline-rich protein 3 | 71.01 | 6 | 8.86 |
| 106 | 63087903 | 231 | 24256 | Unnamed protein product (Trypsin-3) | 8.66 | 1 | 7.46 |
| 107 | 699577 | 338 | 38405 | Lumican | 6.51 | 2 | 6.17 |
| 108 | 71296879 | 78 | 8918 | GPM6A protein | 20.51 | 1 | 6.03 |
| 109 | 7245805 | 497 | 56516 | Chain D, Erythrocyte Catalase | 5.63 | 1 | 6.95 |
| 110 | 74730663 | 189 | 20681 | Plasma cell-induced resident endoplasmic reticulum protein | 24.87 | 3 | 5.22 |
| 111 | 7770227 | 218 | 22725 | PRO2743 (Alpha-2-HS-glycoprotein) | 18.81 | 2 | 5.43 |
| 112 | 82407456 | 113 | 13086 | Chain L, Calprotectin(S100A9) | 41.59 | 4 | 5.71 |
| 113 | 86279007 | 73 | 8127 | Small proline rich protein | 23.29 | 1 | 9.04 |
| 114 | 93116916 | 380 | 42672 | Cytochrome b | 3.16 | 1 | 7.83 |

Table footnote:

^a^. UniProt descriptions for proteins annotated as Unnamed at NCBI has been added in parenthesis with underlined text. These were found using the ID mapping tool at the UniProt web-site.
